# The long and still uncertain journey of BRAF as a prognostic tool in patients with papillary thyroid cancer

**DOI:** 10.20945/2359-3997000000140

**Published:** 2019-05-13

**Authors:** Rafael Selbach Scheffel, Ana Luiza Maia

**Affiliations:** 1 Universidade Federal do Rio Grande do Sul Departamento de Farmacologia Instituto de Ciências Básicas da Saúde Universidade Federal do Rio Grande do Sul Porto Alegre RS Brasil Departamento de Farmacologia, Instituto de Ciências Básicas da Saúde, Universidade Federal do Rio Grande do Sul, Porto Alegre, RS, Brasil; 2 Universidade Federal do Rio Grande do Sul Hospital de Clínicas de Porto Alegre Faculdade de Medicina Universidade Federal do Rio Grande do Sul Porto Alegre RS Brasil Unidade de Tireoide, Hospital de Clínicas de Porto Alegre, Faculdade de Medicina, Universidade Federal do Rio Grande do Sul, Porto Alegre, RS, Brasil

The principle underlying personalized medicine is that using specific information about a person’s disease will help in establishing a diagnostic, a therapeutic strategy or defining a prognosis. Nevertheless, the underlying assumptions of the precision-medicine model may not be obvious, since it includes the existence of meaningful subgroups of patients and the ability to identify them with this approach. Perhaps the most successful area of personalized medicine is cancer treatment. Tumors arise as a result of an accumulative series of events from genetic variants, usually in a limited set of genes, which identification led to molecular-target therapies. Examples of successful use of personalized medicine include the use of targeted therapies to treat specific types of cancer cells, such as HER2-positive breast cancer cells, BRAF mutation‐related melanoma, and RET driven tumors (1).

BRAF is one of the most studied genes in papillary thyroid cancer (PTC). BRAF mutations activate the mitogen-activated protein kinase (MAPK) pathway resulting in increases of cellular proliferation, inhibition of differentiation and apoptosis (2). The BRAF point mutation T1799A is the most common, resulting in the exchange of valine to glutamate at residue 600 (BRAF^V600E^). The first studies describing the role of BRAF^V600E^ mutation on the pathogenesis of PTC were published in 2003 (3). Subsequent studies focused on searching an association between BRAF mutation and features of an unfavorable course of the disease. The impact of the mutation in clinical outcomes (persistent disease, recurrence, survival) remains debatable with studies showing conflicting results (4). So far, it seems that BRAF^V600E^ mutation is not an independent prognostic marker and should be analyzed in association with other prognostic factors.

In this edition of the *Archives of Endocrinology and Metabolism,* two studies shed light on the prognostic role of BRAF^V600E^ on Brazilian patients. Pessôa-Pereira and cols retrospectively studied 43 PTC patients to evaluate the association between BRAF^V600E^ mutation and several clinicopathological features of PTC, meaning tumor size, histological subtype, multifocality, extrathyroidal extension, vascular invasion, lymph node and distant metastases, and AJCC/TNM or recurrence risk stage systems (5). The authors report a high prevalence of BRAF^V600E^ mutation (65.1%), which was associated with older age and absence of Hashimoto’s thyroiditis, but not with other high-risk features. In the study of Giorgenon and cols (6), the authors evaluated BRAF^V600E^ mutation and TERT (telomerase reverse transcriptase) gene promoter mutation preoperatively in 45 high-risk thyroid nodules with confirmed anatomopathological diagnostic PTC. TERT is involved in cellular immortality, by preserving the telomere length at the end of chromosomes and enhancing other cellular functions such as proliferation and cell cycles. The authors also found a high prevalence of BRAF^V600E^ mutation (66.7%), but only 4 patients (8.9%) showed TERT mutation. No association was observed between BRAF^V600E^ mutation and clinicopathological aspects. Interestingly, however, in agreement with previous studies (7), the TERT mutation was associated with advanced age and advanced AJCC TNM stage.

These studies show interesting similarities: both of them observed a high prevalence of BRAF^V600E^ mutation, conflicting with previous studies in South-eastern region (28.1 to 48.3%) (8,9) but in accordance with results obtained from Goiânia (63.8%) and Porto Alegre (59.4%) (10,11). These observations indicate a significant heterogeneity on the prevalence of BRAF^V600E^ mutation among the different regions in Brazil. Contrasting with other reports (4), the studies failed to demonstrate an association between the BRAF^V600E^ mutation and high-risk clinicopathological features, which might be partially explained by the relatively small sample sizes of both studies. Unfortunately, the lack of data on patient-centered outcomes (i.e., status of the disease on long term follow-up) in both studies, and the absence of multivariate analyses on the second one (6) limit the study’s conclusions.

The use of specific molecular classifier as prognostic markers is a research trend in thyroid cancer, and a potential role of BRAF^V600E^ mutation has been long advocated. However, until now, the studies have failed to demonstrate its use as a single, independent predictive factor in clinical practice. Accordingly, the current guidelines do not indicate a routine application of *BRAF* mutation status for initial risk stratification in PTC. Thus, the real place of BRAF^V600E^ as a tool in personalized medicine in the prognostic of PTC patients remains uncertain. On the other hand, studies point that identification *TERT* promoter mutation in preoperative specimens might be useful as an independent marker for risk stratification and helpful on defining the best treatment strategy for PTC patients.
